# Behavioral economic and wellness-based approaches for reducing alcohol use and consequences among diverse non-student emerging adults: study protocol for Project BLUE, a randomized controlled trial

**DOI:** 10.1186/s13063-024-08009-9

**Published:** 2024-03-09

**Authors:** James G. Murphy, Ashley A. Dennhardt, Jacob Tempchin, Hannah E. Colgonis, Meghan E. McDevitt-Murphy, Brian Borsari, Kristoffer S. Berlin

**Affiliations:** 1 Department of Psychology, The University of Memphis, 400 Innovation Dr, Memphis, TN 38152 USA; 2 Mental Health Service (116B), San Francisco VAHCS, San Francisco, CA USA; 3 Department of Psychiatry and Behavioral Sciences, University of California, San Francisco, San Francisco, CA USA

**Keywords:** Behavioral economics, Substance-free activities, Brief motivational intervention, Emerging adulthood, Alcohol misuse

## Abstract

**Background:**

Emerging adults (EAs) who are not 4-year college students nor graduates are at elevated risk for lifetime alcohol use disorder, comorbid drug use, and mental health symptoms, compared to college graduates. There is a need for tailored brief alcohol intervention (BAI) approaches to reduce alcohol risk and to facilitate healthy development in this high-risk population. Most BAIs include a single session focused on discussing risks associated with drinking and correcting normative beliefs about drinking rates. EAs may benefit from additional elements that enhance general wellness. The substance-free activity session (SFAS) aims to clarify life goals and values and increase goal-directed activities that provide alternatives to alcohol use, and the relaxation training (RT) session teaches relaxation and stress reduction skills.

**Methods:**

The present study is a randomized 3-group (BAI + SFAS vs. RT + SFAS vs. education control) trial with 525 EAs (175 per group; estimated 50% women and 50% African American) who report recent risky drinking and who are not students or graduates of 4-year colleges. Participants will have the option of completing the intervention sessions in person or via a secure video teleconference. Levels of drinking and alcohol-related problems will be evaluated at baseline and 1, 3, 6, and 12 months post-intervention. The primary hypothesis is that both BAI + SFAS and RT + SFAS participants will report significantly greater reductions in alcohol use and problems relative to education control participants, with no differences in outcomes between the two active treatment conditions.

**Discussion:**

The results of this study will inform alcohol prevention efforts for high-risk community dwelling emerging adults.

**Trial registration:**

ClinicalTrials.gov Identifier: NCT04776278.

**Supplementary Information:**

The online version contains supplementary material available at 10.1186/s13063-024-08009-9.

## Background

There have been substantial increases in alcohol use, heavy episodic drinking (≥ 4/5 drinks on one occasion for females/males, respectively), alcohol use disorders (AUD), and alcohol-related deaths in the United States over the past decade, particularly among vulnerable subpopulations, including individuals with minoritized racial/ethnic identities as well as individuals with lower educational attainment and family income [1, 2]. Emerging adults (EAs; ages 18–29) have higher levels of recent heavy-episodic drinking (35.6%) and AUD (26.7%) than other age groups [1]. This is not surprising because EAs display a neuro-developmentally mediated tendency towards elevated reward-seeking, impulsivity, and negative affect [3, 4]. Patterns of frequent heavy episodic drinking often lead to significant acute and chronic health and social consequences that can disrupt critical developmental tasks including educational attainment and career advancement and can result in elevated alcohol use across the lifespan [5, 6]. Results from the 2023 Monitoring the Future study suggest that an increasing number of Americans display persistent risky drinking throughout their 30s and 40s, [7] which highlights the importance of disseminating targeted alcohol harm prevention interventions to emerging adults who engage in risky drinking.

Approximately 18.3 million EAs (59% of EAs) in the United States are not current college students [8] and only 41% of EAs will ultimately earn a 4-year college degree. The college environment is protective against many alcohol-related consequences (e.g., driving while intoxicated, arrests) [9], and non-student EAs typically work and live in less enriched and more stressful psychosocial environments than college students. Moreover, failure to earn a 4-year college degree reduces lifetime job stability, status, and salary, which in turn limits the availability of alternative reinforcers and may reduce constraints against drinking throughout adulthood [10, 11]. An analysis of data from the National Survey on Drug Use and Health indicated that non-student EAs report slightly higher levels of heavy episodic drinking than same-age EAs who are college students (40% vs. 37%) [12]. Other epidemiological data suggest that, relative to college graduates, adults without a college degree consume more drinks per occasion and have elevated lifetime risk for AUD/SUD [13].

College student EAs have shown recent population-level reductions in drinking, which may be due in part to steadily increasing university-based prevention efforts over the past two decades, including brief alcohol interventions (BAIs) [14, 15]. BAIs are delivered using a motivational interviewing style (MI) [16] and typically include peer-referenced normative comparisons, personalized feedback on drinking patterns and associated risks, and harm reduction strategies. BAIs have led to significant reductions in drinking across numerous clinical trials with college students and are a Tier 1 prevention approach for college student drinking, resulting in wide dissemination in university settings [14]. The efficacy of these prevention efforts is encouraging [17], but the disproportionate focus on 4-year college students may inadvertently exacerbate existing health and economic disparities between them and individuals without bachelor’s degrees [18, 19]. Thus, it is critically important to provide BAIs for non-student EAs.

To date, most of the BAI trials with non-student EAs have been conducted in medical or employment settings. Reviews of BAIs in EA drinkers suggest that these interventions are more efficacious than no treatment or minimal treatment control conditions [15, 17] and a meta-analysis of BAIs for non-student EAs found that studies implementing counselor-administered MI had statistically significant, though small, effects on alcohol use (*d* = 0.20 [0.04, 0.36]) relative to active controls [20]. Although these are important settings, many EAs do not present in these settings for treatment. Thus, in order to increase generalizability and potential for widespread dissemination across other settings, there is a need to evaluate the efficacy of BAIs among diverse samples of EAs recruited from the community. Moreover, it is essential to evaluate interventions that address the unique risk factors experienced by EAs, including increased negative affect and limited access to substance-free activities [20, 21].

Two recent trials have examined BAIs that were adapted for non-student EAs recruited from communities in the USA. One study evaluated a single-session BAI that included personalized drinking feedback along with strategies for coping with stress and a discussion about participants’ vocational and educational aspirations (*N* = 164, ages 18–25) [22]. There were significant treatment effects on drinking but not alcohol problems at a 3-month follow-up and no drinking reductions at the 6- or 9-month follow-ups. Another study examined the short-term effects of a BAI for non-student EAs recruited from the community who did not plan to attend college (*N* = 167, ages 17–20) [23]. The single-session intervention included standard BAI content (personalized alcohol feedback delivered in a MI style) enhanced with a discussion of vocational and personal goals along with personalized feedback on time allocation to alcohol vs. other activity categories (cf. [24]). One- and three-month follow-up assessments revealed significant reductions in alcohol use and problems relative to a relaxation training control session. Treatment effects were partially mediated by increases in seeking alternatives to drinking [25].

Despite the promise of single session BAIs, effect sizes are generally small and there is a need to enhance these interventions with novel content to increase their efficacy with higher-risk EAs [21, 26]. The modest overall response may be due in part to the limited focus of BAIs, which generally target motivation to reduce drinking without addressing reasons for drinking (e.g., boredom, stress, lack of goals or future orientation) or providing alternative means of experiencing reward or reducing negative affect. Indeed, predictors of poor response to BAI include low levels of substance-free reinforcement, poor self-regulation/impulsivity, low future time orientation, and anxiety or depressive symptoms [24, 27–30].

Furthermore, help-seeking for alcohol-related interventions in EAs is low. One study found that only 23% of EAs were interested in responsible alcohol use, whereas > 45% expressed interest in programs focused on improving mood and planning for the future (careers, money, health, life balance) [31]. Thus, programs that address alcohol use indirectly in the context of an overall focus on wellness and enhancing mood and goal setting might have greater appeal and potential for dissemination than traditional brief alcohol interventions [21, 32].

A recent daily diary study with non-student EA drinkers indicated that engagement in alternative enjoyable activities was the most frequently endorsed strategy to avoid drinking [33]. The substance-free activity session (SFAS) was developed to supplement alcohol-focused BAIs and uses MI, personalized normative feedback, goal setting, and an episodic future thinking exercise to target the behavioral economic mechanisms of substance-free reinforcement and delayed reward discounting [34]. The SFAS encourages EAs to identify and discuss the long-term benefits associated with their life goals and to consider how their current patterns of time allocation and drinking (which are aggregated as personalized feedback) might impact those valued goals [24, 35, 36].

The two-session BAI + SFAS approach has demonstrated efficacy for reducing both alcohol use/problems and depressive symptoms in two randomized controlled trials with college EAs and may be a more promising approach than single-session BAIs for higher-risk non-student EAs. Our first trial [28] found that a two-session (BAI + SFAS) intervention resulted in larger reductions in heavy drinking and alcohol problems than a two-session (BAI + RT) at 6-month follow-up in a sample of 82 college students who reported recent heavy drinking.

A subsequent two-site randomized controlled clinical trial compared BAI + SFAS and BAI + RT to an assessment control condition [24]. The combination of a BAI plus either the SFAS or RT was associated with significant reductions in alcohol use and problems across the 16-month follow-up compared with assessment only. Increases in proportional reinforcement from substance-free activities and protective behavioral strategies mediated treatment effects. Both active treatments were also associated with more global benefits, including significant increases in self-regulation and reductions in depression and anxiety [24]. There were no overall group differences between BAI + SFAS and BAI + RT, although a secondary analysis indicated an advantage for BAI + SFAS for participants with low levels of environmental reward at baseline [37]. Thus, the primary results provide support for both SFAS and RT as potentially efficacious supplements to BAI that may contribute to both drinking reductions and increased overall wellness.

## The present study

The primary goal of the proposed study is to establish the efficacy of the BAI + SFAS and BAI + RT approaches, relative to an education only control condition, among high-risk community-dwelling EAs. The primary hypothesis is that at 1, 3, 6, and 12-month follow-ups both BAI + SFAS and RT + SFAS participants will report significantly greater reductions in alcohol use and alcohol problems relative to education control participants, with no differences in outcomes between the two active treatment conditions. A secondary hypothesis is that the predicted advantage for BAI + SFAS and RT + SFAS, relative to education control, will be mediated by changes in alcohol reinforcing efficacy, negative affective symptoms, and proportionate substance-related reinforcement. An additional secondary hypothesis is that the BAI + SFAS and the RT + SFAS conditions will be associated with greater reductions in alcohol demand, proportionate substance-related reinforcement, drug use, and negative affect than the control condition, with no differences in outcomes between the two active treatments.

## Method

### Design overview

We will conduct a 3-group randomized controlled parallel group superiority (BAI + SFAS vs. education control; SFAS + RT vs. education control) and non-inferiority trial (BAI + SFAS vs. SFAS + RT). Participants will be 525 individuals between the ages of 18 and 29 years old (estimated 50% women and 50% Black) recruited from the Memphis community and via online recruitment efforts across the U.S. Eligible criteria are described below.

### Participants and setting

Participants will be recruited via community events, flyers, and digital and print media that invite “young adults who drink alcohol” to participate in a confidential research study. Additionally, targeted community-based recruitment of African American EAs will be accomplished through recruiting at African American churches, theaters, and cultural events as well as in businesses, restaurants, barber/beauty shops, and community centers located in predominantly African American neighborhoods. Sessions will be conducted either in-person at the University of Memphis or virtually via teleconferencing software.

### Enrollment, consent, randomization, and research assessments (survey) procedures

People interested in participating in the study will complete a brief online screening survey that includes the screening consent form, contact information, and the demographic and drinking items required to determine eligibility for the full study. They will have a 1 in 100 chance of winning a $50 gift card for completing the screening survey. Participants will be eligible for the study if they are ages 18–29, report recent hazardous drinking, as evidenced by two or more past-month heavy drinking episodes (≥ 5/4 drinks for men/women) and/or exceeding NIAAA guidelines for high-risk drinking (> 14/7 drinks per week for men/women), and will not be current full-time 4-year college students or graduates or plan to enroll as full-time 4-year students in the coming year. Eligible participants must also report stable domicile and contact information, speak English fluently, and have a ≥ 9th grade reading ability. Exclusion criteria are current/past psychosis, current self-initiated AUD/SUD treatment, ≥ 3 days per week use of illegal drugs (including misuse of prescription drugs) other than cannabis, and high risk for alcohol withdrawal based on recent drinking pattern and endorsement of withdrawal symptoms.

Participants will complete the baseline assessment via a web-based survey (either remotely or in the research laboratory) and will be asked to provide consent for the full study. Randomization will occur algorithmically immediately after the baseline assessment and will be stratified by gender and race/ethnicity. We will use an urn procedure to ensure baseline treatment group equivalence on past-month frequency of heavy drinking and drug use. Participants randomized to the education condition will receive the educational materials described below immediately after completing the assessment in the context of a 5-min discussion with a study clinician. Participants randomized to either of the active treatments will complete the first 50-min intervention session (BAI or RT) immediately after the baseline assessment. All participants will receive a $50 payment after completing this first phase, with an additional $10 bonus for attending session 1 on time to encourage attendance. Participants in the active treatment conditions will complete the 50-min SFAS 1 week later and will be compensated $40. Following the SFAS, participants will receive weekly, personally tailored text-message delivered support over the 4-week interval. Research staff will send participants appointment reminders by text message and email the day before their appointments. Participants who miss an appointment will be contacted by text message, phone call, and email up to three times per week until their appointment window closes. If we receive no response, we will contact the participant’s alternate contact person.

Research outcomes will be assessed via web-based surveys at 1 month, 3 months, 6 months, and 12 months after the baseline assessment (either remotely or in the research laboratory). See Table 1 for full schedule. Participants will receive a $40 payment for each of the follow-ups that they complete (maximum total study compensation is $260).

**Table 1:** SPIRIT figure—schedule of enrollment, interventions, and assessments

	**Enrollment**	**Baseline assessment and randomization**				**Follow-up**			
			**Session 1**	**Session 2**	**Booster texts**				
**Timepoint**	***-t***_***1***_	***t***_***0***_	***t***_***0***_	***t***_***0***_** + *1w***	***t***_***0***_** + *2w–5w***	***t***_***0***_** + *1 m***	***t***_***0***_** + *3 m***	***t***_***0***_** + *6 m***	***t***_***0***_** + *12 m***
**Enrollment:**									
**Eligibility screen**	X								
**Informed consent**	X	X							
**Allocation**		X							
**Interventions:**									
***Education control***			X			X	X	X	X
***BAI* + *SFAS***			X	X	X	X	X	X	X
***RT* + *SFAS***			X	X	X	X	X	X	X
**Assessments:**									
Demographics	X	X							
**Primary outcomes**									
Daily Drinking Questionnaire		X				X	X	X	X
Past month binge drinking		X				X	X	X	X
Drug Use Questionnaire		X				X	X	X	X
Brief Young Adult Alcohol Consequences Questionnaire		X				X	X	X	X
**Secondary outcomes**									
*Substance-related problems*									
AUD Symptom Checklist		X				X	X	X	X
SUD Symptom Checklist		X				X	X	X	X
Alcohol-Impaired Driving Behavior		X				X	X	X	X
Marijuana Problem Scale		X				X	X	X	X
*Behavioral economic measures*									
Activity Level Questionnaire		X				X	X	X	X
The Alcohol Purchase Task		X				X	X	X	X
Delay discounting		X				X	X	X	X
*Aversive emotional states and coping*									
The Depression, Anxiety, and Stress Scales		X				X	X	X	X
The Brief COPE									
*Drinking and drinking norms*									
NIAAA Drinking Assessment		X							X
Drinking Norms Rating Form		X				X	X	X	X
Protective Behavioral Strategies Survey		X				X	X	X	X
*Personal values*									
Valued Living Questionnaire		X				X	X	X	X

## Interventions

### Brief Alcohol Intervention (BAI)

The alcohol-focused BAI is modeled after the efficacious Brief Alcohol Screening and Intervention for College Students (BASICS) approach [14] that we have included in the trials described above [24]. The 50-min BAI will include a decisional balance exercise, feedback on drinking and drug use patterns, and discussion of drinking norms, alcohol-related consequences, alcohol/drug interactions, alcohol goal setting, and harm-reduction strategies.

### Relaxation training (RT)

The RT session is identical to what we included in the trials with college student participants described above [24, 28] and begins with the clinician providing the rationale that relaxation strategies can reduce stress and enhance wellness and might contribute to drinking reductions. The clinician then leads the participant through a diaphragmatic breathing exercise, a progressive muscle RT protocol, and then a brief breath-counting (mindfulness) exercise. The session will conclude with a brief discussion of additional stress and anxiety management strategies and apps. Participants will be asked about their reaction to the techniques and, if interested, encouraged to commit to a plan for practicing the techniques.

### Substance-free activity session (SFAS)

The SFAS uses an MI plus feedback approach to increase the salience of the individual’s goals, to highlight the connection between their current patterns of behavior (including drinking and substance-free activities) and the attainment of these goals, and to increase future orientation and engagement in enjoyable and goal-directed activities that are inconsistent with alcohol/substance use (even if the participant has no desire to change their use). The specific content of the SFAS session varies according to participants’ unique goals and interests. The clinician makes an effort to focus on activities known to be associated with drinking reductions and mood enhancement, including developing hobbies, strengthening social support, engaging with community/service or religious activities, and exercising or engaging in other wellness-related activities. Participants receive personalized information on locally available and free/inexpensive activities and resources. Text boosters are included to enhance follow-through with goals and to provide activity suggestions.

### Education control condition

This minimal contact control condition will include a brief (~ 5 min) discussion where the research assistant (RA) who completed the assessment session will describe the educational handout. This condition is meant to approximate a public health-level approach to providing referral information and some of the content included in the BAI + SFAS condition but without MI or personalized information. Participants will receive information on risks associated with alcohol/drug misuse, strategies for reducing alcohol problems, managing stress, and goal setting. The handout will also include links to hotlines, websites, and apps related to these domains. This condition will not include booster contact.

## Study measures

### Demographics, personal relationships, health, and finances

We will ask several questions about participants’ demographics and background, including education and employment status, current living arrangement, and relationship status. We will also ask several questions regarding their objective and perceived financial situation as well as how much they spend on non-essentials including alcohol. Health questions will include asking about diagnosed psychological disorders and current treatment for such disorders.

### Primary outcomes

#### Drinking quantity and frequency

The Daily Drinking Questionnaire (DDQ) [38] will be used to measure the quantity and frequency of alcohol use by asking participants to estimate the typical number of drinks consumed on each day of the week during a typical week in the past month. Past-month frequency of binge drinking will be assessed with a single item asking participants to report the number of episodes of consuming ≥ 4/5 drinks in two hours or less for females/males.

#### Alcohol-related negative consequences

The Brief Young Adult Alcohol Consequences Questionnaire (B-YAACQ) [39] is a 24-item self-report measure that assesses whether or not participants have experienced 24 potential alcohol-related negative consequences. The items are summed for a total score (0–24). The B-YAACQ has demonstrated reliability and validity in young adult samples [39].

### Secondary outcomes

#### Drug use

The Drug Use Questionnaire (DUQ) [40] will be used to measure past-month drug use across 7 drug use categories and whether the participant has used the drug(s) at the same time as alcohol.

#### Substance-related problems

The Alcohol Use Disorder Symptom Checklist [41] will be used to assess whether participants are currently experiencing any symptoms, and which symptoms, of an alcohol use disorder. The Substance Use Disorder Symptom Checklist [41] will be used to assess whether participants are currently experiencing symptoms of any non-alcohol substance use disorder. The Marijuana Problem Scale (MPS) [42] will assess marijuana and other drug consequences with a list of 19 potential consequences. Participants report whether the consequences listed have been “no problem,” a “minor problem,” or a “serious problem.”

#### Behavioral economic measures

The modified Activity Level Questionnaire measures past-month activity frequency and enjoyment with Likert scales and separate items for substance-related and substance-free activities [34]. The frequency and enjoyment ratings are multiplied to obtain a cross-product score that reflects reinforcement derived from the activity. The variables of interest will be the average reinforcement from all substance-free activities (substance-free total) and the total reinforcement ratio, i.e., substance-related total/(substance-free total + substance-related total). Reinforcement ratio values have been shown to mediate response to brief alcohol interventions with emerging adults [24]. We modified this measure to also measure time allocation to each activity and perceived constraints on access to the activity. We also ask several open-ended questions about participants’ goals regarding hobbies, service, and community activity participation. This information will be used to provide activity suggestions as part of the SFAS intervention.

The Alcohol Purchase Task (APT) [43] is a simulation measure that assesses self-reported alcohol consumption and financial expenditure across a range of drink prices (alcohol demand). Participants report the number of standard drinks they would consume in a hypothetical drinking scenario across 17 price increments ranging from zero (free) to $20 per drink. Demand curves are estimated by fitting each participant’s reported consumption across the range of prices to Hursh and Silberburg’s [44] demand curve equation: ln *Q* = ln *Q*0 + *k* (*e*-*αQ*0*C* − 1), where *Q* is demand, *Q*0 is the level of demand that occurs when cost, *C*, approaches zero, *K* specifies the range of *Q*, and *α* is a measure of demand curve elasticity. Several demand metrics are generated from the demand curve which reflect individual differences in strength of alcohol as a reinforcer [43], and the proposed analysis will focus on the three indices that have shown the most robust associations with alcohol-related outcomes [34]: (1) intensity of demand (alcohol consumption at the lowest price), (2) Omax (maximum financial expenditure on alcohol), (3) elasticity of demand (sensitivity of alcohol consumption to increases in cost) [34, 43]. Demand curve indices from The Alcohol Purchase Task (APT) will be used as secondary outcomes and mediators of treatment.

Delay discounting (DD) will be measured with a computerized monetary choice questionnaire (ED 50) in which participants repeatedly choose between a larger fixed amount of hypothetical money ($1000) available after a delay and variable smaller amounts of hypothetical money that are available immediately [45].

#### Aversive emotional states and coping

The Depression, Anxiety, and Stress Scales (DASS) [46] will be used to provide feedback to participants in the SFAS condition and to determine whether these symptoms moderate outcomes and change following treatment. The Brief COPE [47] will be used to assess coping at baseline to generate feedback on effective coping resources that will be included in the SFAS. Changes in coping will also be assessed as a secondary outcome variable.

#### Drinking and drinking norms

The NIAAA Drinking Assessment will be used to assess past-year alcohol consumption including drinking frequency, typical number of drinks, maximum number of drinks, and frequency of binge drinking. For each item, participants are given a range of frequencies (e.g., every day to 3–11 times in the past year) or a range of drinks (e.g., 1 drink to 25 or more drinks) to choose from. This measure will be administered at baseline and again at 12 months.

Perceived normative drinking will be assessed with the Drinking Norms Rating Form, and the Protective Behavioral Strategies Survey (PBSS) [48] will be used to measure the use of 15 protective behavioral strategies commonly used to reduce alcohol-related harm. Protective behavioral strategies will also be evaluated as a mediator of treatment outcomes.

#### Personal values

The two-part, 22-item Valued Living Questionnaire [49] assesses both the degree to which a participant values each of eleven domains of life importance (e.g., family relations, spirituality, physical well-being) on a 10-point scale (1 = not at all important, 10 = extremely important) and the degree to which the participant’s past-week actions are consistent with these values on a 10-point scale (1 = not at all consistent, 10 = extremely consistent). Personalized feedback on values and consistent actions is included in the SFAS session.

#### Attention check questions

By providing only one logically correct answer per question (e.g., “To answer this question, please choose option number four”), the five question Conscientious Responders Scale assesses whether participants are attentively answering questions, rather than answering them haphazardly or randomly [50]. We included this scale so study staff would have a clear measure of respondent conscientiousness (4/5 CRS questions answered correctly) and a marker of data quality.

## Study oversight

Oversight of internal monitoring of the participants’ safety will be conducted by the PI, Dr. Murphy. Dr. Murphy has significant experience conducting clinical research of brief alcohol interventions. The Data and Safety Monitoring Plan for this application will begin by implementing standard procedures for day-to-day monitoring of the study by the Investigators and study staff. Investigators Murphy and McDevitt-Murphy and project director Dennhardt will also meet weekly to evaluate the progress of the trial, to review data quality, recruitment, and study retention, and to examine other factors that may affect outcomes. They will also review participant experiences and the rates of adverse events (AEs) to determine any necessary changes to reduce participant risk. Dr. Murphy will be responsible for distinguishing serious from nonserious adverse events and providing attributions (causality and severity). Dr. Murphy will report any serious adverse events (SAEs) in writing within 48 h to the NIAAA Project Officer as defined by NIAAA and to the University of Memphis IRB following their policies. Dr. Murphy will apprise fellow investigators and study personnel of all adverse events that occur during the conduct of this research project through regular study meetings. An annual report will be submitted to the NIAAA project officer summarizing all adverse events. A brief report will be generated quarterly for the study record. If necessary, the investigators will make appropriate recommendations for changes in protocol.

## Interim analyses

In order to preserve power and reduce the likelihood of type 1 error, we will not conduct formal *inferential* interim or futility analysis [51]. Given the nature of interventions we are studying (brief behavioral approaches that have been used previously with no adverse effects) and our target sample (young adults who are not seeking treatment or at risk for alcohol withdrawal), we think that it is extremely unlikely that we would need to stop the trial due to clear evidence of harm or overwhelming evidence of benefit. Adopting an inferential approach to interim analysis would thus unnecessarily undermine our ability to detect our planned moderate effect size outcomes at study conclusion without increasing risk of type one error (if we do not adjust alpha based on interim analyses) or type two error (if we do adjust alpha to accommodate interim analyses).

However, co-investigator and study biostatistician Dr. Berlin will conduct descriptive interim analyses after we have enrolled 75 participants in each condition. The study will be stopped if one of the following three scenarios manifests: (1) there is clear evidence of harm; (2) there is no likelihood of demonstrating treatment benefit (futility); (3) there is overwhelming evidence of the benefit of treatment. Although we do not expect any physical risks with this research, if psychological risks are occurring at a frequency that is higher than expected (more than 5% of participants are experiencing moderate to severe distress or troublesome feelings associated with the questionnaires or interventions), enrollment and interventions will be promptly halted until a safety review is completed by the study investigative team in conjunction with the University of Memphis IRB and the NIAAA Program Officer.

## Statistical analyses

Statistical analyses will be performed using Mplus version 8.4 and SPSS version 26. The distributions of continuous variables including drinking outcomes, mediators, and potential covariates will be examined. Due to the nature of the multi-wave data collection, some participant attrition is inevitable. We will carefully examine the extent and pattern of missing data and will evaluate attrition effects by testing whether systematic differences exist between those who complete all study measures versus those who do not. In instances when a small amount of data is missing from a measure (i.e., < 10%) and relevant missing data correlates are included, data will be assumed to be missing at random, and a robust maximum likelihood estimator will be used (MLR) [52]. The MLR estimator adjusts standard errors and chi-squares/loglikelihoods to accommodate missing data, non-normal distributions, and nesting by individuals and clinicians. Missing not at random models will also be estimated to determine whether the models are robust to the missing at random assumption of maximum likelihood estimation [52, 53]. In the evaluation of intervention outcomes, we will use intention-to-treat analysis. In our prior studies using similar intervention procedures 99% of participants completed all intervention sessions [28, 54].

### Preliminary analyses

Preliminary data analyses will include studies of patterns of missing data, dropout rates, therapist adherence, distributional properties of measures, and correlations among outcome measures. Outliers greater than 3.29 standard deviations (SDs) above or below the mean will be recoded to one unit above or below the highest or lowest nonoutlier value [55]. Second, we will use latent growth curve modeling (LGCM) to model trajectory shapes and assess growth rates over time and individual differences in the growth rates as a function of intervention. While the first approach will allow us to examine if and when the effects start to decay, the LGCM approach will inform us of the overall pattern of changes across time and allow us to test if the trajectory shapes differ between the control and the intervention conditions. With five measurement time points, we will be able to estimate trajectory shapes accurately beyond a straight linear trajectory. Linear and non-linear trajectory will be investigated to accurately model the trajectory shape supporting the hypotheses on intervention effects, as the outcomes may not decrease constantly as a function of time. As such, we will investigate the declining or plateauing measurement time points and model non-linear trajectory (e.g., piecewise, quadratic growth).

### Intervention outcome analyses

Consistent with our previous approaches [24], evaluation of our intervention outcomes will occur via generalized linear mixed models with a negative binomial distribution and log link function (due to our expectation that the primary drinking outcomes will be skewed and zero-inflated). These models will (A) evaluate change in the primary drinking outcomes (drinks per week , past-month binge drinking, and alcohol-related problems) and (B) evaluate change in secondary outcomes (increases in proportionate reinforcement from substance-free activities and self-regulation, reductions in alcohol demand, drug use, and negative affect) across the four follow-up time points (1-, 3-, 6-, and 12-month) as a function of time and treatment condition. Time will be centered at the 1-month follow-up (time = 0 at 1-month follow-up) and baseline levels of the outcome will be included as a covariate. Random slope effects (i.e., time-by-treatment interactions) will be explored; however, our previous trial found they were not significant and were eliminated from the final reported models. Thus, we anticipate that final models will be estimated using a compound symmetry covariance matrix with random intercepts and linear effects of time. Age, gender, and ethnicity will be included as covariates due to their association with drinking level and the possibility of differential treatment response [56]. Models will be run with the following planned contrasts: active treatment versus education control, BAI + SFAS versus education control, RT + SFAS versus education control, and BAI + SFAS versus RT + SFAS. To test the hypothesis that no differences in outcomes will be found between the two active treatments, the two one-sided test (TOST) procedure for testing equivalence/noninferiority will be used [57]. Based on this approach, equivalence is established at the α significance level if a (1–2α) × 100% confidence interval for the difference in efficacies (RT + SFAS—BAI + SFAS) is contained within the equivalence margin or the interval (-δ, δ). This equivalence margin, *δ*, reflects the range of values for which treatments are “close enough” to be considered equivalent [57]. We have chosen the value *δ* = 1.12, for alcohol use, and *δ* = 0.82 problems, based on 75% the lower limit of a confidence interval of the difference between the BAI + SFAS against the assessment only in our previous trial [57–59].

### Exploratory moderation analyses

The potential moderating effects of race (White vs. Black), sex, income (above. vs. below federal poverty level), past-month illicit drug use, and elevated negative affective symptoms (scoring above threshold on the DASS stress, anxiety, or depression scales) will be explored via path analyses in a manner analogous to multiple regression procedures [60]. The dependent variable in question will be regressed on the main effects for intervention condition, the baseline values of the dependent variable, the baseline moderator variable, and the interaction between the intervention condition and the moderator, where a significant interaction indicates that the effects of the independent variables differ at levels of the moderator. A significant interaction will be followed up with simple slopes analyses at high (i.e., one SD above the mean or clinical cut-off) and low (i.e., one SD below the mean or clinical cut-off) moderator values that will determine the nature of the moderated effect. All continuous variables will be centered prior to the tests for moderation. Variables that directly predict outcome but do not interact with intervention condition will be retained as covariates.

### Mediator analyses

We hypothesize that the predicted advantage for BAI + SFAS and RT + SFAS, relative to control, will be mediated by reductions in alcohol demand, negative affect (DASS total score) and increases in proportionate substance-free reinforcement. Mediators will be tested in separate multilevel (mixed) mediation models using the product of coefficients approach [61]. This approach provides an estimate of the mediated effect by multiplying the regression of the mediator on the independent variable (the “a-path”), and the regression of the outcome on the mediator (the “b-path”), with the independent variable included in the model [62]. The 95% confidence intervals for the mediated effects will be estimated using RMediation [63]. All models will be estimated in Mplus Version 8.4122 with robust maximum likelihood estimation that adjusts standard errors and chi-squares/loglikelihoods to accommodate missing data, non-normal distributions, and nesting by individuals and clinicians. Missing not at random models will also be estimated to determine whether the models are robust to the missing at random assumption of maximum likelihood estimation [52]. Finally, we will conduct exploratory differential (moderated) mediation models that test the hypothesis that alcohol demand (directly targeted by the BAI) will have a larger indirect effect on changes in alcohol use and problems in the BAI + SFAS than in the RT + SFAS condition and that negative affect (targeted by both the RT + SFAS sessions) will have a larger indirect effect on changes in alcohol use and problems in the RT + SFAS condition than in the BAI + SFAS condition.

## Sample size and power

We have focused our power analysis using Monte Carlo simulations [64] with population estimates and patterns of missingness from our previous trial [24]. These models from our previous trial used generalized mixed models which covaried gender and race/ethnicity, taking into account clustering by individual and therapist. Based on the Monte Carlo estimates comparing BAI + SFAS to control (within *d* = 0.83/0.71, between *d* = 0.33/0.32), we are proposing to recruit 525 subjects (175 per group) for the study. Assuming a similar pattern of attrition as our previous trial will result in *n* = 362 (69%) complete cases, *n* = 95 (18%) cases missing one data point; *n* = 37 (7%) cases missing two data points; *n* = 21 (4%) cases missing three data points; and *n* = 11 (2%) cases missing four data points. We calculated power via Monte Carlo simulations by determining the proportion of 1000 replications for which the null hypothesis that a parameter was equal to zero was rejected for each parameter at alpha = 0.05 (i.e., probability of rejecting the null hypothesis when it is false). This resulted in the following power estimates for typical drinks/problems: treatment effect of BAI + SFAS vs. control = 1.00/1.00; mediation via proportionate substance-free reinforcement = 0.90/0.99; mediation via negative affect = 0.85/0.93; mediation via alcohol demand: 1.00/1.00. While our proposed sample size would not be sufficient to detect very small effects, we believe such small effects are likely to have little clinical meaning in terms of the variables we are assessing in the present trial, and the necessary sample size to detect such effects would be very large (> 1000).

## Harms

The project director and the principal investigator (PI) will be responsible for overseeing AE and SAE reporting. RAs will take initial reports from participants, and the project director and PI will review for safety and accuracy and recommend follow-up as needed. All AEs and SAEs will be reviewed within five business days. Any SAEs possibly related to study intervention will be reported to the IRB and funding agency within five business days.

## Confidentiality

Data will be collected through participant self-reports completed via computerized assessments and recordings of individual counseling sessions (for treatment fidelity analyses). All data will be collected in secure and private laboratory settings or via a secure, encrypted Zoom call by trained graduate student research assistants/clinicians who have completed university and lab specific training in human subject protections and non-judgmental interviewing techniques. All data are for research purposes and only for the proposed project. The host of the computerized assessment, Rivulent, Inc., contracts with a secure data center that meets industry-standard SAS 70, SSAE 16, and SOC requirements. This ensures physical security protocols including cameras in all sections of the facility, keycard access to server rooms, and locked server cabinets to prevent unauthorized access, even among data center personnel. All data collected is protected in transit by utilizing industry-standard 2048-bit encrypted SSL certificate to ensure data cannot be gleaned through a “man -in-the-middle” style attack. Data is stored within a secure, protected database system, and all personally identifiable information (PII) is stored encrypted using the widely adopted and heavily vetted AES-128-CBC algorithm, which is also the designated encryption method for the United States Government. The server implements industry-standard firewall protection. Following the completion of the assessments, all data will be stored on a secure server stored in the University of Memphis Psychology Department. Our servers are on the university network secured behind our firewall and require user authentication per user to access. The University of Memphis Single Sign-On System (SSO) now includes multi-factor authentication (MFA) capabilities. This means that users can protect their accounts by requiring a second means to authenticate in addition to their password. Participants will be allocated unique trial identification numbers and study data will be associated only with these numbers.

## Data access

Study data will initially be available only to the study Investigators and their labs. After the investigators have published the main outcomes of the trial, they will provide public access to a de-identified data set, consistent with the NIH Policy for Data Management and Sharing.

## Auditing

While the funder does not plan regular audits, the study team is prepared for any possible audits from the funder or the approving IRB.

## Dissemination policy

The research team will disseminate the results of this study through the publication of peer-reviewed manuscripts. All contributing authors will be appropriately credited. In accordance with NIH public access policy, published results will be made publicly available at the NIH National Library of Medicine’s PubMed Central immediately after journal publication. In addition, at the completion of data analysis, all study participants will be e-mailed a copy of a lay summary of the results.

## Discussion

The proposed study has the potential to provide important information on the efficacy of two different brief alcohol intervention approaches with high-risk emerging adults who are not four-year college students or graduates. Notably, there is strong evidence for the efficacy of brief alcohol interventions with college students, but very few studies have explicitly evaluated these approaches with ethnically diverse samples of emerging adults who are not college students (Davis et al., 2017). Moreover, this is the first trial to evaluate two session approaches with this higher risk group, including sessions primarily focused on enhancing substance-free activities and reducing negative affect (RT + SFAS). If effective, RT + SFAS may have greater potential for dissemination than BAI approaches that are generally not used by EAs without sanctions or incentives and that require potentially stigmatizing alcohol screening questions [65, 66]. Should the RT + SFAS successfully reduce alcohol use and negative affective symptoms and increase positive activities it could be disseminated as a brief wellness and goal setting program in a variety of community settings (high schools, vocational training programs, churches, health or community centers) where it could benefit a large population of EAs. Importantly, EAs could benefit from RT + SFAS even if they do not drink or if they do not want to change their use, which should reduce stigma and enhance potential for dissemination.

## Trial status

Recruitment for this trial began on December 8, 2021. We anticipate enrollment to be completed by the end of June 2025. At the time this manuscript is being written, protocol version 4.0 from May 16, 2022, is in use.

### Protocol amendments

All protocol amendments will be submitted to the IRB for approval. The funder will approve all major protocol changes before implementation.

### Reproducible research

At the completion of this study, its protocol will be published on Clinical-Trials.gov, and the corresponding dataset and statistical code will be available upon request to the principal investigator. This manuscript uses SPIRIT reporting guidelines [67]. A SPIRIT checklist is included as an additional file.

### Supplementary Information

**Additional file 1:** Model consent form.

**Additional file 2. **

## Data Availability

External researchers may submit a request to the investigative team to obtain access to this data for secondary analysis and publication. Data will also be submitted to NIAAA Data Archive (NIAAA_DA_), a data repository housed within the NIMH Data Archive (NDA).

## Abbreviations

AE: Adverse event
APT: Alcohol purchase task
AUD: ALCOHOL use disorder
B-YAACQ: Brief Young Adult Alcohol Consequences Questionnaire
BAI: Brief alcohol intervention
DASS: Depression, Anxiety, and Stress Scales
DD: Delay discounting
DDQ: Daily Drinking Questionnaire
DUQ: Drug Use Questionnaire
EA: Emerging adult
IRB: Institutional Review Board
LGCM: Latent growth curve modeling
MI: Motivational Interviewing
NIAAA: National Institute on Alcohol Abuse and Alcoholism
NIH: National Institutes of Health
PI: Principal investigator
RA: Research assistant
RT: Relaxation training
SAE: Serious adverse event
SD: Standard deviation
SFAS: Substance-free activity session
