# Supplementary material for: Behavioral economic and wellness-based approaches for reducing alcohol use and consequences among diverse non-student emerging adults: study protocol for Project BLUE, a randomized controlled trial
Source: Trials. 2024 Mar 9;25:173. doi: 10.1186/s13063-024-08009-9 (PMC10924404; doi:10.1186/s13063-024-08009-9)
Supplement: Supplementary file 1 — Additional file 1: Model consent form. [file 13063_2024_8009_MOESM1_ESM.docx]

**Appendix 1: Model Consent Form**

**1. KEY INFORMATION:**

**Voluntary Consent:** You are being asked to volunteer for a research study. It is up to you whether you choose to participate or not. There will be no penalty or loss of benefit to which you are otherwise entitled if you choose not to participate or discontinue participation.

**Purpose:** The purpose of this study is to evaluate the effects of three approaches for improving young adult adjustment and reducing risky alcohol consumption.

**Duration:** It is expected that your participation will last 1 year.

**Procedures and Activities:** You will be asked to complete several questionnaires related to your lifestyle, drinking and things that happen when you drink or use drugs, and your attitudes regarding drinking and other activities. This session should take approximately 50 minutes. Then, you will be assigned to one of three groups. In two of the conditions, you will complete either a) a one-on-one conversation about your drinking including receiving individualized feedback about your drinking and risks or b) a session that will teach you about relaxation techniques immediately after you complete the questionnaires. This session would take approximately 50 minutes. (Total time today – Consent + 50 minutes of questionnaires + 50-minute intervention = ~2 hours). You will return in approximately 1 week and have another individual meeting that would entail having a conversation about your lifestyle and goals. You will receive a text-message with information on how to follow-through with your goals weekly for 4 weeks after the second session. A third condition involves receiving information on low-risk drinking strategies, managing stress, and goal setting in a 2–5-minute conversation with our study staff that would occur today immediately after you complete the study questionnaires. You will not receive text messages following this session. We do not know whether one of these approaches is more helpful than the other. Immediately after each session, you will complete a short survey that will allow you to rate the session. Follow-up assessments will be held 1, 3, 6 and 12 months from now. During these sessions, you will complete the same questionnaires related to your lifestyle, drinking and things that happen when you drink, drug use, and your attitudes regarding drinking and other activities.

**Risk:** Some of the foreseeable risks or discomforts of your participation include loss of confidentiality due to the nature of the sensitive information you provide to us. We will make every effort to ensure your privacy and protect the information that you give us.

**Benefits:** Some of the benefits that may be expected include that you may learn more about your alcohol and drug use.

**Alternatives:** As an alternative to participation, we could provide you with resources for obtaining information on alcohol and drug use**.**

**2. STUDY DESCRIPTION:**

**Who is conducting this research?**

Dr. James Murphy of the University of Memphis, Department of Psychology is in charge of the study. There may be other research team members assisting during the study. No member of the research team has a significant financial interest, and/or a conflict of interest related to the research.

**Why is this research being done?**

The purpose of this study is to evaluate the effects of three approaches for improving young adult adjustment and reducing risky alcohol consumption. You are being invited to participate because you indicated you have consumed alcohol in the past month and are between the ages of 18-29.

**How long will I be in this research?**

The research will be conducted at the University of Memphis. It should take about 5-7 hours total of your time depending on which condition you are assigned to. Your first session will take 1-2 hours followed by a 1-hour session a week later depending on condition assignment. All participants will then be invited to participate in 4 1-hour sessions that will take place at 1, 3, 6 and 12 months after your initial appointment.

**What happens if I agree to participate in this Research?**

If you agree you will be asked to complete several questionnaires related to your lifestyle, drinking and things that happen when you drink or use drugs, and your attitudes regarding drinking and other activities. You may skip any question that makes you uncomfortable and you can stop any time. This session should take approximately 50 minutes. Then, you will be placed in one of three conditions by chance (Like a coin toss/like drawing straws). You have a 1 in 3 chance of being placed in any of the study groups. You cannot pick your group. In two of the conditions, you will complete either a) a one-on-one conversation about your drinking including receiving individualized feedback about your drinking and risks or b) a session that will teach you about relaxation techniques immediately after you complete the questionnaires. This session would take approximately 50 minutes. (Total time today – Consent + 50 minutes of questionnaires + 50-minute intervention = ~2 hours). You will return in approximately 1 week and have another individual meeting that would entail having conversation about your lifestyle and goals. You will receive a text-message with information on how to follow-through with your goals weekly for 4 weeks after the second session. A third condition involves being provided with information on low-risk drinking strategies, managing stress, and goal setting immediately after you complete the study questionnaires today. This will take ~ 5 minutes and you will not receive text-messages if you are in this condition. We do not know whether one of these approaches is more helpful than the other. Immediately after each session, you will complete a short survey that will allow you to rate the session. The group you are assigned to is a matter of chance. You will be given $50 for today’s appointment and $40 for completing the appointment next week.

Follow-up assessments will be held 1, 3, 6 and 12 months from now. During these sessions, you will complete the same questionnaires related to your lifestyle, drinking and things that happen when you drink, drug use, and your attitudes regarding drinking and other activities. You will receive $40 for each of the 4 follow-ups that you complete. After your 12-month assessment, you will be given the option to complete any of the intervention sessions that you did not complete as a part of the study or to complete a session for a second time.

In order for this project to have scientific value, we need to know whether our intervention was helpful.

Therefore, we will make every effort to contact you for these follow-up interviews. As part of your participation in this project, we will ask your permission to contact another person who knows you well enough to know how to contact you over the next six months. We will not inform any individual about the nature of research study or speak with them about any of the confidential material you have given us as part of this study.

Audio recordings will be made of these sessions for supervisory purposes, so that we can check to make sure the project procedures are being implemented as planned. In addition, we plan to study parts of the interventions that are related to changes in alcohol use. This information will help us further improve future interventions. These audio recordings will be identified only by an identification number and will be stored separately from all other information. Any personal information provided during the session (e.g., name of friends, classes attended) will be purged from transcripts made of the sessions. In addition, any information on the audiotapes is protected by the Certificate of Confidentiality obtained for this study (see section 7). These audiotapes and transcripts will be destroyed following their use in this study.

As a participant, you have the right to request the results (which study condition had the best results) when we have completed analyzing the data using the researcher’s contact information on the first page of this document.

**What happens to the information collected for this research?**

The information collected for this research will be used to publish results in journal articles and conference presentations, but we will keep your name and other identifying information confidential.

This study is funded by the National Institute of Alcohol Abuse and Alcoholism, and thus we are required to abide by their data sharing expectations Data from this study will be submitted to the National Institute of Mental Health Data Archive (NDA) at the National Institutes of Health (NIH). NDA is a large database where deidentified study data from many National Institute of Mental Health (NIMH) studies is stored and managed. Deidentified study data means that all personal information about you (such as name, address, birthdate and phone number) is removed and replaced with a code number. Sharing your deidentified study data helps researchers learn new and important things about mental health and substance use more quickly than before.

During and after the study, the study researchers will send deidentified study data about your health and behavior to the NDA. Other researchers across the world can then request your deidentified study data for other research. Every researcher (and institution to which they belong) who requests your deidentified study data must promise to keep your data safe and promise not to try to learn your identity. Experts at the NIH who know how to keep your data safe will review each request carefully to reduce risks to your privacy. Sharing your study data does have some risks, although these risks are rare. Your study data could be accidentally shared with an unauthorized person who may attempt to learn your identity. The study researchers will make every attempt to protect your identity.

You may not benefit directly from allowing your study data to be shared with NDA. The study data provided to NDA may help researchers around the world learn more about mental health and substance use and how to help others who have problems with mental health and substance use. NIMH will also report to Congress and on its website about the different studies using NDA data. You will not be contacted directly about the study data you contributed to NDA.

You may decide now or later that you do not want your study data to be added to the NDA. You can still participate in this research study even if you decide that you do not want your data to be added to the NDA. If you know now that you do not want your data in the NDA, please tell the study researcher before leaving today. If you decide any time after today that you do not want your data to be added to the NDA, call or email the study staff who conducted this study, and they will tell NDA to stop sharing your study data. Once your data is part of the NDA, the study researchers cannot take back the study data that was shared before they were notified that you changed your mind. If you would like more information about NDA, this is available on-line at <http://nda.nih.gov>.

U.S. Law requires that a description of this clinical trial be available on the website [www.clinicaltrials.gov](http://www.clinicaltrials.gov). This study has been registered as a clinical trial ClinicalTrials.gov and results information will be submitted as required according to current policy. The website will not include information that can identify you. The website will only include a summary of this study.

**How will my privacy and data confidentiality be protected?**

We promise to protect your privacy and security of your personal information as best we can. Although you need to know about some limits to this promise. Measures we will take include:

All data will be kept in locked file cabinets or password protected files on lab computers.

Your name will be removed from the file that contains your answers to the survey questions and replaced with an ID number.

We have obtained a Certificate of Confidentiality (CoC) from the National Institutes of Health (NIH). The CoC has been issued to protect the investigators on this study from being forced to tell people that are not connected with this study about your participation in this study, even under a subpoena. The protection offered by the CoC does not stop us from voluntarily reporting information about suspected or known sexual, physical, or other abuse of a child or older person, threats of violence to self or others, threats to destroy property or serious communicable diseases. If any member of the research team is given such information, he or she will make a report to the appropriate authorities.

Even when a CoC is in place, you and your family members must still continue to actively protect your own privacy. If you voluntarily give your written consent to an insurer, employer, or lawyer to receive information about your participation in the research, then we may not use the CoC to withhold this information. The research record will include documents that contain private information about your health and other behaviors.

All data will be obtained specifically only for research purposes. All data will be kept confidential and not directly linked to any individual participant. Contact and identifying information will be collected for booster and follow-up purposes but will be kept separate from the actual data. Contact and identifying information (e.g., name, address, phone number, email address, date of birth, name of school you attend) will be destroyed after completion of last follow-up or if you do not complete the last follow up, within six-months of study enrollment. However, if you consent separately to be contacted for future research study opportunities, your contact information will be kept for up to 10 years for that purpose. All self-report data will be collected online with every effort made to have participants complete all measures on a secure web-based server. NIH, the study’s funding agency, has an open data sharing agreement, meaning that study data sent to NIH will never be destroyed. At the conclusion of this study, the study data shared with NIH will be archived and de-identified, meaning that it will not include any contact or identifying information (e.g., name, address, phone number, email address, date of birth, name of school you attend).

By law, there are a few limits to confidentiality. These limits were developed in part to ensure the safety of research participants. The researchers are required by law to take some action if there is suspicion that you may harm somebody else or there is suspicion that a child may be in danger. If any of these situations should occur, we will attempt to contact you prior to taking any action.

Individuals and organizations that monitor this research may be permitted access to inspect the research records. This monitoring may include access to your private information. These individuals and organizations include the University’s Institutional Review Board.

**What if I want to stop participating in this research?**

It is up to you to decide whether you want to volunteer for this study. It is also ok to decide to end your participation at any time. There is no penalty or loss of benefits to which you are otherwise entitled if you decide to withdraw your participation. If you choose to withdraw from the study, we will retain previously obtained deidentified data unless you request that it be deleted/destroyed. Your decision about participating will not affect your relationship with the researcher(s) or the University of Memphis.

**Will it cost me money to take part in this research?**

- There are no costs associated with participation in this research study.

The University of Memphis does not have funds set aside to pay for the cost of any care or treatment that might be necessary because you got hurt or sick while taking part in this study. Also, the University of Memphis will not pay for any wages you may lose if you are harmed by this study.

**Will I receive any compensation for participating in this research?**

- You will receive $50 for today’s appointment and $40 for each additional study appointment that you attend for a total of $250. You can choose to be paid via cash, Venmo, or Cash App.
  - The online survey contains attention checks and if you fail the attention checks you will not be compensated.
  - Please be aware compensation for participation in research may be considered taxable income. The University may require tracking for compensation that is paid to you; this may include your name and contact information. This information is stored confidentially and separate from research data.

**Who can answer my question about this research?**

Before you decide to volunteer for this study, please ask any questions that might come to mind. Later, if you have questions, suggestions, concerns, or complaints about the study, you can contact the investigator, Dr. James Murphy at 901-871-9976. If you have any questions about your rights as a volunteer in this research, contact the Institutional Review Board staff at the University of Memphis at 901-678-2705 or email [irb@memphis.edu](mailto:irb@memphis.edu). We will give you a signed copy of this consent to take with you.

In certain circumstances (such as being a suicide risk), the study administrator will have the right to terminate your participation in the study. If termination was deemed appropriate, the study administrator would make every effort to contact you directly and provide you with information on mental health resources in the community.

**STATEMENT OF CONSENT**

I have had the opportunity to consider the information in this document. I have asked any questions needed for me to decide about my participation. I understand that I can ask additional questions through the study.

By signing below, I volunteer to participate in this research. I understand that I am not waiving any legal rights. I have been given a copy of this consent document. I understand that if my ability to consent for myself changes, my legal representative or I may be asked to consent again prior to my continued participation.

As described above, you will be Audio recorded while performing the activities described above. Audio recording will be used to ensure the quality of each session. Initial the space below if you consent to the use of audio recording as described.

____ I agree to the use of audio recording.

As described above, your data (but not personal information such as name, address, birthdate, and phone number) will be entered into the National Institute of Mental Health Data Archive (NDA).

____ I agree to sharing my data with NDA.

|  |  | | |  |  |  |
| --- | --- | --- | --- | --- | --- | --- |
| **Name of Adult Participant** | |  | **Signature of Adult Participant** | |  | **Date** |
|  |  | | |  |  |  |

**Researcher Signature (To be completed at the time of Informed Consent)**

I have explained the research to the participant and answered all of their questions. I believe that they understand the information described in this consent and freely consent to participate**.**

|  |  | | |  |  |  |
| --- | --- | --- | --- | --- | --- | --- |
| **Name of Research Team Member** | |  | **Signature of Research Team Member** | |  | **Date** |
